# Semisupervised Deep State-Space Model for Plant Growth Modeling

**DOI:** 10.34133/2020/4261965

**Published:** 2020-05-25

**Authors:** S. Shibata, R. Mizuno, H. Mineno

**Affiliations:** ^1^Graduate School of Integrated Science and Technology, Shizuoka University, 3-5-1 Johoku, Naka-ku, Hamamatsu, Shizuoka 432-8011, Japan; ^2^College of Informatics, Academic Institute, Shizuoka University, 3-5-1 Johoku, Naka-ku, Hamamatsu, Shizuoka 432-8011, Japan; ^3^JST, PRESTO, 4-1-8 Honcho, Kawaguchi, Saitama 332-0012, Japan

## Abstract

The optimal control of sugar content and its associated technology is important for producing high-quality crops more stably and efficiently. Model-based reinforcement learning (RL) indicates a desirable action depending on the type of situation based on trial-and-error calculations conducted by an environmental model. In this paper, we address plant growth modeling as an environmental model for the optimal control of sugar content. In the growth process, fruiting plants generate sugar depending on their state and evolve via various external stimuli; however, sugar content data are sparse because appropriate remote sensing technology is yet to be developed, and thus, sugar content is measured manually. We propose a semisupervised deep state-space model (SDSSM) where semisupervised learning is introduced into a sequential deep generative model. SDSSM achieves a high generalization performance by optimizing the parameters while inferring unobserved data and using training data efficiently, even if some categories of training data are sparse. We designed an appropriate model combined with model-based RL for the optimal control of sugar content using SDSSM for plant growth modeling. We evaluated the performance of SDSSM using tomato greenhouse cultivation data and applied cross-validation to the comparative evaluation method. The SDSSM was trained using approximately 500 sugar content data of appropriately inferred plant states and reduced the mean absolute error by approximately 38% compared with other supervised learning algorithms. The results demonstrate that SDSSM has good potential to estimate time-series sugar content variation and validate uncertainty for the optimal control of high-quality fruit cultivation using model-based RL.

## 1. Introduction

Several studies have been performed to evaluate advanced cultivation techniques for stable and efficient production of high-quality crops based on farmers' experience and intuition [1–4]. For example, water stress cultivation of tomato plants is a technique that increases their sugar content by reducing irrigation. The technique requires sensitive irrigation control to provide the appropriate water stress throughout the cultivation period. A fine balance must be achieved because insufficient water stress does not improve sugar content while excessive water stress causes permanent withering. Such a technique is currently limited to expert farmers, and there have been some studies conducted to estimate water stress indirectly from soil moisture or climatic environmental factors such as temperature, humidity, and sunlight [5–10]. Recent studies have attempted to assess water stress with deep neural networks by monitoring plant motion caused by withering [11, 12]. Those studies contributed to the quantification of water stress to improve water stress cultivation to some extent. However, the purpose of water stress cultivation is to raise the sugar content, and a technique to directly control the sugar content flexibly is of interest. In this regard, our final goal is to develop a method to determine the optimal action to achieve the desired sugar content of greenhouse tomato plants at harvest stably and efficiently. In this study, we aim to develop a plant growth model to estimate time-series sugar content variation employing reinforcement learning, as the first step toward the final goal.

Reinforcement learning (RL) [13–15] acquires an optimal strategy through the experience of an agent performing an action in an environment and has demonstrated high flexibility and nontask-dependent representation capabilities. There are two types of RL: model-free RL [16, 17] and model-based RL [18, 19]. Model-free RL does not use the environmental information (state transition of the environment) to predict how the environment changes and what type of reward is obtainable when the environment is modified. By contrast, model-based RL uses the information of the state transition of the environment. Therefore, model-based RL is better than model-free RL at judging behavior based on a long-term plan with regard to a future state. Namely, model-based RL is expected to perform well in determining optimal actions to achieve the desired sugar content at harvesting.

The training of model-based RL involves two steps: (1) modeling an environment and (2) planning to learn the optimal policy for the model. In this paper, we focus on modeling an environment and developing a plant growth model for the optimal control of water stress cultivation. The model-based RL's environmental model is often a probabilistic model evaluated based on the standard deviation, because the plant states and the surrounding environment data are time-series data and generally contain a significant amount of noise [20]. The noise is affected not only by external factors but also by internal factors such as nonlinearly distributed plant growth. For example, nondestructively measured sugar content data based on spectroscopy varies depending on the location of the measurement point on a fruit because of the uneven internal structure of a fruit. Thus, we need to select a robust model that can properly handle such noisy data.

Among probabilistic models, the generative model is known as the most suitable for plant growth modeling, because generative models assign low probability to outliers. By contrast, discriminative models process the training dataset without considering the effects of noise. The generative model not only is robust to noise but also has good characteristics for predicting future states [20] and for generalization performance [21].

When model-based RL is used for the optimal cultivation of high sugar content fruits, it is necessary to predict future plant conditions from the cultivation environment based on present conditions. Moreover, it is important that the modeling method can be applied to different plant varieties and specific environments as well as to various environments. Therefore, we use the generative model to achieve high generalization performance of plant growth modeling, which requires predictability of the future states. In particular, we try to make the generative models much more robust and flexible by using sequential deep generative models combined with a state-space model (SSM, a typical generative model for time-series data) and because deep neural networks have fewer restrictions.

The variational autoencoder (VAE) [22] is a deep generative model for nonsequential data, and the parameters are optimized via stochastic gradient variational Bayes (SGVB) [23]. The stochastic recurrent networks (STORN) [23] are highly structured generative processes that are difficult to fit to deterministic by combining the elements of the VAE. Additionally, STORN is able to generate high-dimensional sequences such as music by including recurrent neural networks (RNN) in the structure of VAE and represents stochastic sequential modeling by inputting a sequence independently sampled from the posterior to a standard RNN. The variational RNN (VRNN) was proposed by Chung et al. [24] as a model similar to STORN. The main difference is that the prior of the latent variable depends on all previous information via a hidden state in the RNN. The introduction of temporal information has been shown to help in modeling highly structured sequences. VAE is also applied for optimal control. Watter et al. [25] addressed the local optimal control of high-dimensional nonlinear dynamic systems. Considering optimal control as the identification of the low-dimensional latent space, their proposed model Embed to Control (E2C) is trained while compressing high-dimensional observations such as images. Their results showed that E2C exhibits strong performance in various control tasks. Krishnan et al. [26] modeled the change of a patient's state over time using temporal generative models called Deep Kalman Filters (DKF). Unlike previous methods, DKF incorporates action variables to express factors external to patients, such as prescribing medication or performing surgery. In particular, structured variational inference is introduced in DKF to cater to the unique problems of living organisms, such as considering that a patient's states vary slowly and that external factors may have long-term effects on patients. These phenomena are similar to plant growth modeling.

The methods described above require a comparatively large-scale dataset to model complex generative processes. On the other hand, creating a large-scale dataset for the time-series sugar content of fruits is not temporally or financially easy because it is necessary to use a sensor to make gentle contact with the fruit manually. In addition, because measurements are performed manually, it is necessary to establish the methods based on various considerations such as measurement time, position, repetition of measurements, and measurement of the surrounding tomatoes to reduce the variance of the measurement value. By contrast, it is common to measure the fruit juice of representative fruits at the time of harvest (destructive measurement). Once the destructive measurement is performed, it is not possible to measure the same fruit over time. For these reasons, the data collection interval required for sugar content is longer than that of automatically sensed data such as temperature, humidity, and solar radiation. In particular, creating a large-scale dataset for time-series crop condition and quality is also not easy using manual measurements because of the workload and cost factors. Thus, it is important to develop a suitable method even though the amount of available data is not large.

In this study, we propose a novel sequential deep generative model called a semisupervised deep state-space model (SDSSM) to evaluate such defective data. SDSSM is similar to DKF in that a deep neural network is used for enforcement of the representation capability of SSM. On the other hand, the major difference is that SDSSM is trained by semisupervised learning to achieve a balance between high generalization performance and high representation power, even if some types of training data are sparse.

## 2. Materials and Methods

### 2.1. Plant Growth Model

#### 2.1.1. Overview of SDSSM

**Figure 1:**
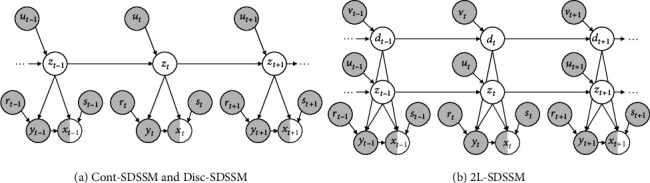
Graphical representations of proposed models.

Based on the general SSM, we assume the following generative processes for plant growth modeling:

(1) $$\begin{array}{c} p_{\theta }\left(z_{t}\;|\;z_{t-1},u_{t}\right)=N\left(z;\mu_{\text{z}}\left(z_{t-1},u_{t}\right),\sigma_{z}\left(z_{t-1},u_{t}\right)\right),\left(\text{system model}\right), \end{array}$$

(2) $$\begin{array}{c} p_{\theta }\left(x_{t}\;|\;z_{t},{\text{s}}_{t}\right)=N\left(x;\mu_{\text{y}}\left(z_{t},s_{t}\right),\sigma_{\text{y}}\left(z_{t},s_{t}\right)\right),\left(\text{observation model}\right), \end{array}$$

where *z*_*t*_ and *x*_*t*_ are latent variables and observed variables, respectively, at time step *t*. The probabilistic models are shown in Figure 1. In our task, the latent variable *z*_*t*_ denotes the plant states. We assume that the water content and the duration of plant growth, which are particularly strongly related to sugar content, are plant states.

Regarding these two states as single-type states with continuous variation, we set a normal distribution to the latent variables *z*_*t*_ according to the previous studies of deep generative models, where a normal distribution was adopted for continuous values. The observed variables *x*_*t*_ indicate the sugar content and are assumed to follow a normal distribution considering the continuous variation of sugar content. Moreover, we introduce the action variables *u*_*t*_, *r*_*t*_, and *s*_*t*_ to the system model and observation model, respectively.

The action variable *u*_*t*_ is added to the process of the state transition considering that plant states vary according not only to previous states but also with external factors such as temperature. In fact, the accumulated temperature is well-known in the agricultural domain as the growth indicator for plants. The action variable *s*_*t*_ is added to the process of the emission because sugar is produced via photosynthesis based on a plant's state and its surrounding environmental variables such as carbon dioxide (CO_2_) concentration. The detailed settings of each random variable are discussed in Section 2.2.2.

Training the generative model based on Equations (1) and (2) using SGVB requires a large amount of data owing to the assumption of the complex generative process: there are implicitly two types of states in the single latent space, and the state transition and emission of the observation are strongly nonlinear. Deep generative models trained by semisupervised learning have recently demonstrated significantly improved generalization capabilities in comparison to previous methods and perform very well even for very small datasets [21, 27, 28].

In particular, conditional VAE (CVAE) is a typical deep generative model trained by semisupervised learning, and the generative model and learning algorithm are based on VAE. VAE learns the parameters simultaneously with the inference of the latent states using only observations. On the other hand, CVAE introduces labels for observations as latent variables to improve the quality of prediction by exploring information in the data density. However, CVAE does not assume missing observations. To explore missing observations efficiently, we take a different approach to CVAE by applying a probabilistic model of SDSSM, as shown in Figure 1(a). Formally, we assume the following generative model:

(3) $$\begin{array}{c} p_{\theta }\left(z_{t}\;|\;z_{t-1},u_{t}\right)=N\left(z;\mu_{\text{z}}\left(z_{t-1},u_{t}\right),\sigma_{z}\left(z_{t-1},u_{t}\right)\right),\left(\text{system model}\right),\\ p_{\theta }\left(x_{t}\;|\;z_{t},{\text{s}}_{t}\right)=N\left(x;\mu_{x}\left(z_{t},s_{t}\right),\sigma_{x}\left(z_{t},s_{t}\right)\right),\left(\text{observation model}\right),\\ p_{\theta }\left(y_{t}\;|\;z_{t},r_{t}\right)=N\left(y;\mu_{y}\left(z_{t},r_{t}\right),\sigma_{y}\left(z_{t},r_{t}\right)\right),\left(\text{observation model}\right), \end{array}$$

where *y*_*t*_ is an additional observed variable that follows a normal distribution and is generated from the same latent variable *z*_*t*_ as is *x*_*t*_, and *r*_*t*_ is the action variable added to the emission of the observation *y*_*t*_. Thus, we assume that observation *y*_*t*_ is a generative process similar to observation *x*_*t*_. The difference between observations appears through the nonlinear functions having different forms and inputs. In particular, sharing latent variables allows one to infer the latent states complementarily to the other observations, even when one observation is missing. Therefore, SDSSM learns the complex latent space as efficiently as other deep generative models, even when the training dataset includes few observations. Here, the functions *μ*_*z*_, *σ*_*z*_, *μ*_*y*_,*σ*_*y*_, *μ*_*x*_, and *σ*_*x*_ are arbitrary nonlinear functions parameterized by deep neural networks (DNNs) as follows:

(4) $$\begin{array}{c} \mu_{\text{z}}={\text{DNN}}_{z}\left(z_{t-1},u_{t}\right),\log\sigma_{z}={\text{DNN}}_{z}\left(z_{t-1},u_{t}\right),\\ \mu_{x}={\text{DNN}}_{x}\left(z_{t},s_{t}\right),\log\sigma_{x}={\text{DNN}}_{x}\left(z_{t},s_{t}\right),\\ \mu_{y}={\text{DNN}}_{y}\left(z_{t},r_{t}\right),\log\sigma_{y}={\text{DNN}}_{y}\left(z_{t},r_{t}\right), \end{array}$$

where DNN_*z*_, DNN_*x*_, and DNN_*y*_ are deep neural networks that have weight matrices *w*_*z*_, *w*_*x*_, and *w*_*y*_, respectively. Thus, the parameters of the generative model are *θ* = {*w*_*z*_, *w*_*x*_, *w*_*y*_}. According to Kingma and Welling [29], we assume that *μ*_z_, *μ*_*x*_, and *μ*_*y*_ denote the mean and *σ*_*z*_, *σ*_*x*_, and *σ*_*y*_ indicate a diagonal covariance matrix. To ensure definite positivity, the outputs from deep neural networks for the diagonal covariance matrix are taken using their logarithm.

#### 2.1.2. Learning SDSSM Using SGVB

We maximize the marginal log-likelihood based on the labeled dataset (*D*_*l*_) and the unlabeled dataset (*D*_*u*_) to optimize the parameters *θ* and *φ* in the generative model. The labeled dataset (*D*_*l*_ = {(*x*_*t*_1_^(*l*)^_, *y*_*t*_1_^(*l*)^_, *u*_*t*_1_^(*l*)^_, *s*_*t*_1_^(*l*)^_, *r*_*t*_1_^(*l*)^_) ⋯ (*x*_*t*_*m*_^(*l*)^_, *y*_*t*_*m*_^(*l*)^_, *u*_*t*_*m*_^(*l*)^_, *s*_*t*_*m*_^(*l*)^_, *r*_*t*_*m*_^(*l*)^_)}) does not include missing values, whereas the unlabeled dataset (*D*_*u*_ = {(*y*_*t*_1_^(*u*)^_, *u*_*t*_1_^(*u*)^_, *s*_*t*_1_^(*u*)^_, *r*_*t*_1_^(*u*)^_) ⋯ (*y*_*t*_*n*_^(*u*)^_, *u*_*t*_*n*_^(*u*)^_, *s*_*t*_*n*_^(*u*)^_, *r*_*t*_*n*_^(*u*)^_)}) includes missing values of observations *x*_*t*_. Note that the labeled data is *x*_*t*_. Here, the superscript *l* represents labeled and the superscript *u* represents unlabeled. We treat *x*_*t*_ in the labeled dataset as observed variables and *x*_*t*_ in the unlabeled dataset as latent variables. In the following, we omit the dependence of *p* and *q* on *u*_*t*_, *v*_*t*_, *s*_*t*_, and *r*_*t*_. The marginal log-likelihood on the labeled dataset is as follows:

(5) $$\begin{array}{c} \text{Log}p_{\theta }\left(x_{1:T},y_{1:T}\right)=\log\int p_{\theta }\left(x_{1:T},y_{1:T}\;|\;z_{1:T}\right)dz_{1:T}. \end{array}$$

Note that we describe *x*_1_, *x*_2_, ⋯, *x*_*T*_ at each time step *t* (*t* = 1, 2,…, *T*) as *x*_1:*T*_. Following the principle of SGVB, we maximize the evidence lower bound (ELBO) with respect to parameters *θ* and *φ* instead of maximizing the marginal log-likelihood directly. We derive a labeled ELBO *ℒ*^*l*^(*x*, *y*; *θ*, *φ*) by introducing the recognition model into Equation (14) and using Jensen's inequality:

(6) $$\begin{array}{c} \text{Log}p_{\theta }\left(x_{1:T},y_{1:T}\right)\geq \log\int q_{\varphi }\left(z_{1:T}\;|\;x_{1:T},y_{1:T}\right)\frac{p_{\theta }\left(x_{1:T},y_{1:T}z_{1:T}\right)}{q_{\varphi }\left(z_{1:T}\;|\;x_{1:T},y_{1:T}\right)}dz_{1:T}=-L^{l}\left(\text{x},\text{y};\theta ,\varphi \right), \end{array}$$

where *q*_*φ*_ is the posterior approximation of latent variable *z*_*t*_, called the recognition model. In general, SGVB approximates the true posterior without factorization. There is an assumption that the true posterior distribution is factorized to a simpler form using a mean-field approximation in the framework of variational inference. Relaxing the constraint contributes to the improvement of the representation capability.In the case of sequential data, the Kullback–Leibler (KL) divergence terms in ELBO often have no analytic form. The gradients of the KL terms are derived by sampling estimation so that insufficient sampling leads to high-variance estimations [26]. Fraccaro et al. [30] derived low-variance estimators of the gradients using true factorization of the posterior distribution according to the Markov property. On the basis of their work, we factorize the recognition model as follows:

(7) $$\begin{array}{c} p\left(z_{1:T}\;|\;x_{1:T},y_{1:T}\right)=\overset{T}{\underset{t=1}{\prod }}p\left(z_{t}\;|\;z_{t-1},x_{t:T},y_{t:T}\right). \end{array}$$

We set the initial latent state to zero:*z*_0_ = 0. The studies mentioned above derived a similar form, such that a latent state at time step *t* is conditioned by previous latent states and by the sequential observations and action variables from time step *t* to *T*. In our case, the form (including the future sequence of observations *x*_*t*_) cannot be calculated owing to the assumption that the observations are missing. However, Krishnan et al. [26] demonstrated that the sequence from the initial time step 0 to time step *t* contains sufficient information. Drawing on their work, we factorize the recognition model as follows:

(8) $$\begin{array}{c} q_{\varphi }\left(z_{1:T}\;|\;x_{1:T},y_{1:T}\right)=\overset{T}{\underset{t=1}{\prod }}q_{\varphi }\left(z_{t}\;|\;z_{t-1},x_{1:t},y_{1:t}\right). \end{array}$$

Based on the decomposed recognition models, the labeled ELBO *ℒ*^*l*^(*x*, *y*; *θ*, *φ*) is defined as follows:

(9) $$\begin{array}{c} -L^{l}\left(\text{x},\text{y};\theta ,\varphi \right)=\overset{T}{\underset{\text{t}=1}{\sum }}E_{q_{\varphi }\left(z_{t}\right)}\left[\log p_{\theta }\left(y_{t}\;|\;z_{t}\right)+\log p_{\theta }\left(x_{t}\;|\;y_{t},z_{t}\right)\right]-\beta \text{KL}\left(q_{\varphi }\left(z_{1}\right)\left\|p_{\theta }\left(z_{1}\right)\right.\right)-\overset{T}{\underset{t=2}{\sum }}E_{q_{\varphi }\left(z_{t-1}\right)}\left[\beta \text{KL}\left(q_{\varphi }\left(z_{t}\right)\left\|p_{\theta }\left(z_{t}\;|\;z_{t-1}\right)\right.\right)\right], \end{array}$$

where *q*_*φ*_(*z*_*t*_) = *q*_*φ*_(*z*_*t*_ | *z*_*t*−1_, *x*_*t*_, *y*_*t*_). The expectations with respect to *q*_*φ*_(*z*_*t*_) and *q*_*φ*_(*z*_*t*−1_) in Equation (9) are estimated via Monte Carlo sampling after applying the reparameterization trick. KL denotes a Kullback–Leibler divergence. All KL terms in Equation (9) can be computed analytically. Additionally, we add the weight coefficient *β* for the KL divergence and gradually increase it from a small number during training to facilitate flexible modeling by the explicit avoidance of restrictions.In the unlabeled dataset, we are interested in the marginal log-likelihood log*p*_*θ*_(*y*_1:*T*_), which is derived by marginalizing out not only *z*_*t*_ but also *x*_*t*_. We obtain an unlabeled ELBO from the marginal log-likelihood in the same way as we obtained the labeled ELBO. The unlabeled ELBO is decomposed as follows by applying d-separation to the graphical model:

(10) $$\begin{array}{c} \text{Log}p_{\theta }\left(y_{1:T}\right)\geq \iint q_{\varphi }\left(z_{1:T},x_{1:T}\;|\;y_{1:T}\right)\log\frac{p_{\theta }\left(x_{1:T},y_{1:T},z_{1:T}\right)}{q_{\varphi }\left(z_{1:T},x_{1:T}\;|\;y_{1:T}\right)}dz_{1:T}\text{d}x_{1:T}=\iint q_{\varphi }\left(z_{1:T}\;|\;x_{1:T},y_{1:T}\right)q_{\varphi }\left(x_{1:T}\;|\;y_{1:T}\right)\log\frac{p_{\theta }\left(x_{1:T},y_{1:T},z_{1:T}\right)}{q_{\varphi }\left(z_{1:T}\;|\;x_{1:T},y_{1:T}\right)q_{\varphi }\left(x_{1:T}\;|\;y_{1:T}\right)}dz_{1:T}\text{d}x_{1:T}. \end{array}$$

Unlike the labeled dataset, an unlabeled ELBO has two recognition models, *q*_*φ*_(*z*_1:*T*_ | *x*_1:*T*_, *y*_1:*T*_) and *q*_*φ*_(*x*_1:*T*_ | *y*_1:*T*_), because the two latent variables, *z*_*t*_ and *x*_*t*_, are included. The former has the same form as the recognition model of the labeled ELBO. On the other hand, the latter is factorized as follows, following a similar approach to the labeled dataset:

(11) $$\begin{array}{c} q_{\varphi }\left(x_{1:T}\;|\;y_{1:T}\right)=\overset{T}{\underset{t=1}{\prod }}q_{\varphi }\left(x_{t}\;|\;y_{t:T}\right). \end{array}$$

Using this decomposed recognition model, an unlabeled ELBO is eventually defined as

(12) $$\begin{array}{c} -L^{u}\left(\text{x},\text{y};\theta ,\varphi \right)=\overset{T}{\underset{\text{t}=1}{\sum }}E_{q_{\varphi }\left(x_{t}\;|\;y_{t}\right)}\left[-L^{l}\left(\text{x},\text{y};\theta ,\varphi \right)+H\left[q_{\varphi }\left(x_{t}\;|\;y_{t}\right)\right]\right], \end{array}$$

where *H*[*q*_*φ*_(*x*_*t*_ | *y*_*t*_)] denotes the entropy of the recognition model *q*_*φ*_(*x*_*t*_ | *y*_*t*_). The unlabeled ELBO *ℒ*^*u*^(*x*, *y*; *θ*, *φ*) includes the labeled ELBO *ℒ*^*l*^(*x*, *y*; *θ*, *φ*), and all probability models except for the recognition model *q*_*φ*_(*x*_*t*_ | *y*_*t*_) are shared by the labeled ELBO and unlabeled ELBO. An expectation in the unlabeled ELBO is estimated via Monte Carlo sampling from the recognition model *q*_*φ*_(*x*_*t*_ | *y*_*t*_). We derive an objective function by summing the labeled and unlabeled ELBOs as follows:

(13) $$\begin{array}{c} J=\underset{D_{l}}{\sum }L^{l}\left(\text{x},\text{y};\theta ,\varphi \right)+\underset{D_{u}}{\sum }L^{u}\left(\text{x},\text{y};\theta ,\varphi \right)+\alpha E_{D_{u}}\left[-\log q_{\varphi }\left(x_{t}\;|\;y_{t}\right)\right], \end{array}$$

where *α* denotes a small positive constant. Because the recognition model *q*_*φ*_(*x*_*t*_ | *y*_*t*_) has only an unlabeled ELBO, it does not acquire label information during training. In accordance with Krishnan et al. [26], we add a regression term to the objective function to train the recognition model using both the labeled dataset and the unlabeled dataset. The objective function is differentiable owing to the differentiable labeled and unlabeled ELBOs, and the parameters *θ* and *φ* are optimized simultaneously by stochastic gradient descent via back-propagation.

#### 2.1.3. Extension of SDSSM

We propose two additional types of extensions to SDSSM depending on some assumptions of the latent space. We call the SDSSM described above a continuous SDSSM (Cont-SDSSM) to distinguish it from the other two models. There are two main plant states that are strongly related to sugar content.

The first is the growth stage [31]. The products of photosynthesis, such as organic acid and sugar content, are accumulated in the fruit, and the ratio of accumulated components varies depending on the growth stage with the transition in metabolism. Therefore, the growth stage is considered essential for plant growth modeling. Second, the sugar content also varies depending on the water content in the plant because insufficient water content often suppresses photosynthesis. Regarding these complicated state transitions with continuous variation, Cont-SDSSM uses single latent variables that follow a normal distribution. We have to consider the appropriate distribution to model the complex transitions and highly structured generative processes based on a plant's growth and surrounding environmental data.

As another approach for modeling the plant states, we design a simpler model called discrete SDSSM (Disc-SDSSM), which represents only the growth stage without considering the water content. In Disc-SDSSM, the forms of generative model, recognition model, and objective function are the same as those in Cont-SDSSM. The major difference is that the latent states follow a categorical distribution because the growth stage is defined as the plant growth class before the harvest, e.g., the flowering stage and ripening stage, which implies discrete growth. We use a Gumbel-Softmax distribution as the posterior of the latent states *z*_*t*_ (instead of a normal distribution) to obtain differentiable categorical samples via the categorical reparameterization trick [32]. Formally, the generative model is defined as follows:

(14) $$\begin{array}{c} p_{\theta }\left(z_{t}\;|\;z_{t-1},u_{t}\right)=\text{Cat}\left(z;\pi_{\text{z}}\left(z_{t-1},u_{t}\right)\right),\left(\text{system model}\right),\\ p_{\theta }\left(x_{t}\;|\;z_{t},{\text{s}}_{t}\right)=N\left(x;\mu_{\text{x}}\left(z_{t},s_{t}\right),\sigma_{\text{x}}\left(z_{t},s_{t}\right)\right),\left(\text{observation model}\right),\\ p_{\theta }\left(y_{t}\;|\;z_{t},r_{t}\right)=N\left(y;\mu_{y}\left(z_{t},r_{t}\right),\sigma_{y}\left(z_{t},r_{t}\right)\right),\left(\text{observation model}\right), \end{array}$$

where *π*_*z*_ is an arbitrary nonlinear function parameterized by deep neural networks as follows: *π*_z_ = NN_*z*_(*z*_*t*−1_, *u*_*t*_).As a different approach to representing plant states, we design a generative model called two latent SDSSM (2L-SDSSM), as shown in Figure 1(b). 2L-SDSSM uses two types of latent variables, *z*_*t*_ and *d*_*t*_, to clearly separate the two plant states. The generative model is defined as follows:

(15) $$\begin{array}{c} p_{\theta }\left(z_{t}\;|\;z_{t-1},u_{t}\right)=N\left(z;\mu_{\text{z}}\left(z_{t-1},u_{t}\right),\sigma_{z}\left(z_{t-1},u_{t}\right)\right),\left(\text{system model}\right),\\ p_{\theta }\left(d_{t}\;|\;d_{t-1},v_{t}\right)=\text{Cat}\left(d;\pi_{\text{d}}\left(d_{t-1},v_{t}\right)\right),\left(\text{system model}\right),\\ p_{\theta }\left(x_{t}\;|\;z_{t},{\text{s}}_{t}\right)=N\left(x;\mu_{\text{x}}\left(z_{t},s_{t}\right),\sigma_{\text{x}}\left(z_{t},s_{t}\right)\right),\left(\text{observation model}\right),\\ p_{\theta }\left(y_{t}\;|\;z_{t},r_{t}\right)=N\left(y;\mu_{y}\left(z_{t},r_{t}\right),\sigma_{y}\left(z_{t},r_{t}\right)\right),\left(\text{observation model}\right), \end{array}$$

where *z*_*t*_ denotes the water content in the plants, *d*_*t*_ denotes the growth stage, and *v*_*t*_ is an action variable playing the same role as the action variable *u*_*t*_ in Cont-SDSSM. The random variables *z*_*t*_ and *d*_*t*_ are mutually independent latent variables that follow a normal distribution and category distribution, respectively. We sample random latent variables *d*_*t*_ from a Gumbel-Softmax distribution via the categorical reparameterization trick, which is similar to our handling of Disc-SDSSM. The labeled and unlabeled ELBOs have different forms owing to differences in the generative model as follows:

(16) $$\begin{array}{c} -L^{l}\left(\text{x},\text{y};\theta ,\varphi \right)=\overset{T}{\underset{\text{t}=1}{\sum }}E_{q_{\varphi }\left(z_{t}\right),q_{\varphi }\left(d_{t}\right)}\left[\log p_{\theta }\left(x_{t}\;|\;z_{t}\right)+\log p_{\theta }\left(y_{t}\;|\;z_{t}\right)\right]-E_{q_{\varphi }\left(z_{t-1}\right),q_{\varphi }\left(d_{t-1}\right)}\left[\beta \text{KL}\left(q_{\varphi }\left(z_{t}\right)\left\|p_{\theta }\left(z_{t}\;|\;z_{t-1}\right)\right.\right)+\beta \text{KL}\left(q_{\varphi }\left(d_{t}\right)\left\|p_{\theta }\left(d_{t}\;|\;d_{t-1}\right)\right.\right)\right],\\ L^{u}\left(\text{x},\text{y};\theta ,\varphi \right)=\overset{T}{\underset{\text{t}=1}{\sum }}E_{q_{\varphi }\left(x_{t}\right)}\left[-L^{l}\left(\text{x},\text{y};\theta ,\varphi \right)+H\left[q_{\varphi }\left(x_{t}\right)\right]\right], \end{array}$$

where *q*_*φ*_(*z*_*t*_) = *q*_*φ*_(*z*_*t*_ | *z*_*t*−1_, *x*_*t*_, *y*_*t*_), *q*_*φ*_(*d*_*t*_) = *q*_*φ*_(*d*_*t*_ | *d*_*t*−1_, *x*_*t*_, *y*_*t*_), and *q*_*φ*_(*x*_*t*_) = *q*_*φ*_(*x*_*t*_ | *y*_*t*_).

### 2.2. Experiments

#### 2.2.1. Experimental Dataset

**Figure 2:**
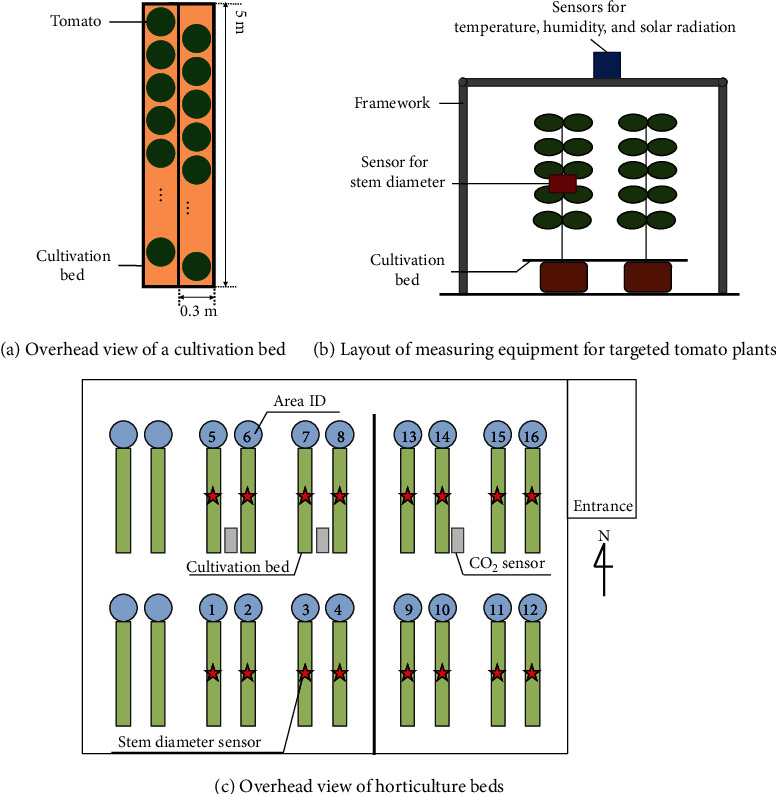
Experimental environment.

We grew tomato plants (*Solanum lycopersicum L.*) in a greenhouse at the Shizuoka Prefectural Research Institute of Agriculture and Forestry in Japan from August 28 to November 18, 2017. There were 16 cultivation beds in the greenhouse, as shown in Figure 2(c), and we cultivated 24 individual tomato plants in each cultivation bed, as shown in Figure 2(a). The tomatoes were grown by three-step dense-planting hydroponic cultivation. Under the three-step dense-planting hydroponic cultivation (Figure 2(b)), the quantity and timing of irrigation greatly affects the quality (sugar content) of tomatoes.

To collect data, we installed sensors in the greenhouse to measure the temperature, humidity, solar radiation, CO_2_ concentration, and stem diameter. The stem diameter was measured by laser displacement sensors (HL-T1010A, Panasonic Corporation) of the target tomato plants. The stem diameter decreases with exposure to high solar radiation in the daytime and increases with decreased exposure to solar radiation in the evening. In addition, the maximum daily stem-shrinkage of the stem diameter is susceptible to a vapor-pressure deficit [33]. All sensor data were collected every minute, and the daily averaged values were used for the model inputs. In addition, we measured the sugar content of the fruits of the target tomato plants on which the stem diameter sensors were installed. The sugar content was measured every three days by a hand-type near-infrared spectrometer (CD-H100, Chiyoda Corporation), and the measurements were conducted in each step of the three-step dense-planting hydroponic cultivation. We used the average sugar content value of 10 time measurements for each fruit in which stem diameter sensors were installed.

#### 2.2.2. SDSSM Variable Settings

The three models (Cont-SDSSM, Disc-SDSSM, and 2L-SDSSM) used for the estimation of sugar content are analyzed through comparative evaluations. This is to reveal the performance of the proposed SDSSM. The variables for the proposed models are listed in Table 1. In this paper, each variable of the proposed three models is set as listed in Table 1; *x*_*t*_ is sugar content, *y*_*t*_ is stem diameter; vector *u*_*t*_ is temperature, solar radiation, vapor-pressure deficit (VPD), the elapsed date from flowering, and accumulated temperature (which is the total temperature from the flowering date to the present); and vector *s*_*t*_ is the CO_2_ concentration, solar radiation, and step ID of three-step dense-planting hydroponic cultivation to indicate to which step of the tomato the data belong. The action variables *r*_*t*_ are not used in any models in this experiment. The settings of each variable in Disc-SDSSM are similar to those of Cont-SDSSM. The difference is that *v*_*t*_ includes the elapsed date from flowering and the accumulated temperature. 2L-SDSSM has similar settings to Cont-SDSSM, and the differences are that *u*_*t*_ includes temperature, solar radiation, and VPD, while *v*_*t*_ includes the elapsed date from flowering and the accumulated temperature.

**Figure 3:**
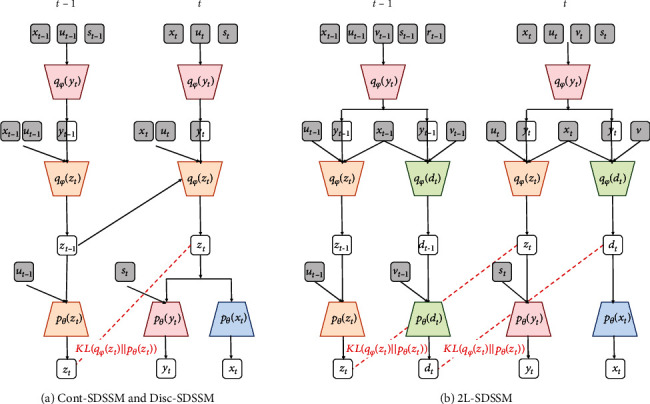
Overview of information flow at time step *t* of the proposed models.

**Figure 4:**
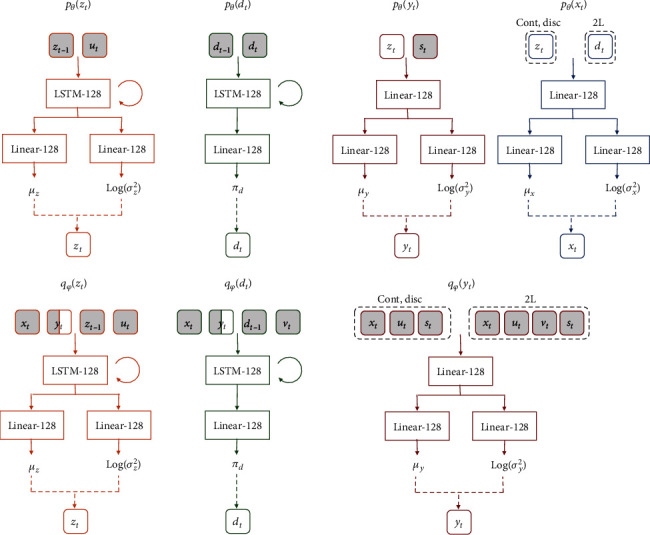
Network architectures showing each neural network in the proposed models.

An overview of the information flow at time step *t* in the graphical model (Figure 1) of the proposed method is shown in Figure 3 by using probability distributions. In addition, Figure 4 shows the inputs and outputs of each probability distribution for the neural network architectures. Cont-SDSSM and Disc-SDSSM include five types of neural networks corresponding to five types of probability distributions: *p*_*θ*_(*x*_*t*_), *p*_*θ*_(*y*_*t*_), *p*_*θ*_(*z*_*t*_), *q*_*φ*_(*x*_*t*_), and *q*_*φ*_(*z*_*t*_). 2L-SDSSM has seven types of neural networks corresponding to its seven types of probability distributions: *p*_*θ*_(*x*_*t*_), *p*_*θ*_(*y*_*t*_), *p*_*θ*_(*z*_*t*_), *p*_*θ*_(*d*_*t*_), *q*_*φ*_(*x*_*t*_), *q*_*φ*_(*z*_*t*_), and *q*_*φ*_(*d*_*t*_).

These seven types of neural networks have the same basic architecture: a hidden layer converts the input nonlinearly, and then the outputs are converted to a mean vector and diagonal covariance log-parameterization matrix through a single separate hidden layer. The neural networks have hidden layers structured as follows: fully connected layers, rectified linear unit (ReLU) layers [34], and batch normalization layers [35]. The neural networks that emit latent states *z*_*t*_ or *d*_*t*_ use a long short-term memory (LSTM) [36, 37] as the first hidden layer instead of a fully connected layer. In this case, LSTM has a forget gate, input gate, and output gate, which makes it possible to consider long-term time series. Therefore, LSTM is used as the first hidden layer. All hidden layers have 128 units, and all weights are initialized using He et al.'s initialization [38] to accelerate convergence. All random variables of Cont-SDSSM follow a normal distribution. On the other hand, the random latent variable*d*_*t*_is categorically distributed. This is because categorical distribution has only one parameter, *π*; Disc-SDSSM and 2L-SDSSM have two consistent hidden layers without the branch structure used for Cont-SDSSM.

#### 2.2.3. Experimental Conditions

We verified the performance of the proposed methods through two types of evaluations. In the first evaluation, we compared semisupervised SDSSMs to supervised SDSSMs trained using only labeled data via supervised learning to verify the effectiveness of our semisupervised learning approach. In this experiment, the supervised Cont-SDSSM, Disc-SDSSM, and 2L-SDSSM are called Cont-SV (SV denotes supervised), Disc-SV, and 2L-SV, respectively. In addition, the semisupervised Cont-SDSSM, Disc-SDSSM, and 2L-SDSSM are called Cont-SSV (SSV denotes semisupervised), Disc-SSV, and 2L-SSV, respectively.

In the second evaluation, we compared semisupervised SDSSMs with typical deep neural networks, the multilayer perceptron (MLP) and stacked LSTM (sLSTM). This is to investigate the performance of the proposed models for the optimal estimation of sugar content. We expected that the proposed models fit all of the observed data rather than local data because the KL terms in ELBO perform a regularization function. On the other hand, both MLP and sLSTM which were trained on a small dataset can easily result in overfitting. Therefore, we applied dropout [39] to each layer in the MLP and sLSTM to reduce overfitting.

We trained each model using the collected tomato dataset. We divided the dataset into training data, validation data, and test data to train and evaluate the models appropriately. In addition, to validating the robustness of the proposed methods, cross-validation was conducted using data sets of 16 cultivation beds divided into four patterns, as shown in Table 2. The hyperparameters were tuned by using random sampling. The hyperparameter such as learning rate, optimization algorithms, dimension size of latent variables for SDSSMs, sequence length, and dropout rate were the same for all the compared models.

When training SDSSMs, we gradually increased the weight coefficient for the KL terms in ELBO by 0.0001 after each epoch (starting from 0 to 1). The mean absolute error (MAE), root mean squared error (RMSE), relative absolute error (RAE), and relative squared error (RSE) were used as the error indicators. In this study, all the models were tuned using the validation data, and the models which showed the lowest MAE were selected as the best models with the most optimal hyperparameters. In this experiment, we implemented all source codes using Python. We used Chainer [40] to implement a deep neural network architecture and scikit-learn [41] to preprocess the dataset. This evaluation was performed on a PC with an Intel Core i7-5820K Processor, GeForce GTX 1080, and 64 GB of memory. The training process time depends largely on the values of the hyperparameters, the tuning method, and the number of epochs. For example, when we used the training data for pattern C in Table 2, which had the largest number of test labeled contents for training, it took approximately 4.6 s to complete 1 epoch, so it took approximately 1 h to create one model converge. Regarding the inference time, it took approximately 5.33 s to infer the test data in 2L-SDSSM when it was repeatedly measured 100 times using the same test data (pattern C in Table 2).

## 3. Results and Discussion

**Figure 5:**
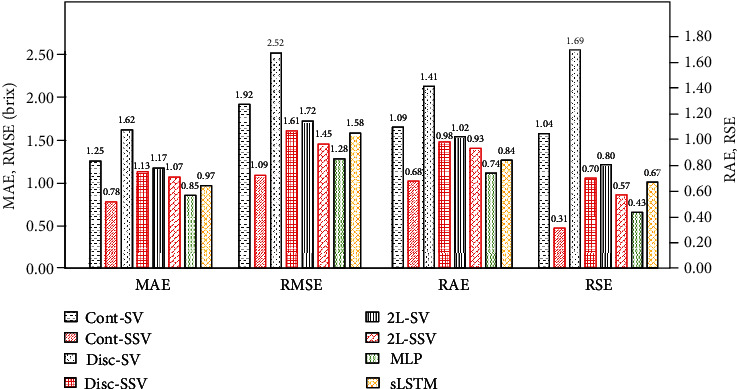
Error indicators of Cont-SV, Cont-SSV, Disc-SV, Disc-SSV, 2L-SV, 2L-SSV, MLP, and sLSTM.

Figure 5 shows the average errors of each supervised SDSSM (Cont-SV, Disc-SV, and 2L-SV), the semisupervised SDSSMs (Cont-SSV, Disc-SSV, and 2L-SSV), MLP, and sLSTM for the four types of test data. The results demonstrate that all semisupervised SDSSMs reduced the estimation errors for all error indicators. In particular, the MAE of Cont-SV is 1.25, whereas that of Cont-SSV is 0.78; the MAE reduction rate of Cont-SSV versus Cont-SV is approximately 38%. Similarly, the MAE reduction rates of the semisupervised SDSSMs versus the supervised SDSSMs are approximately 30% for Disc-SSV and 9% for 2L-SSV. Moreover, both the RAE and RSE of all semisupervised SDSSMs are less than 1. Therefore, all semisupervised SDSSMs perform better than the naive models which output the average of the true values as the estimation values. The MAEs of typical deep neural networks, such as MLP and LSTM, are larger than those of Cont-SSV and less than those of Disc-SSV and 2L-SSV. Although the error indicators of MLP and sLSTM appear to better than other models, further analysis is needed.

**Figure 6:**
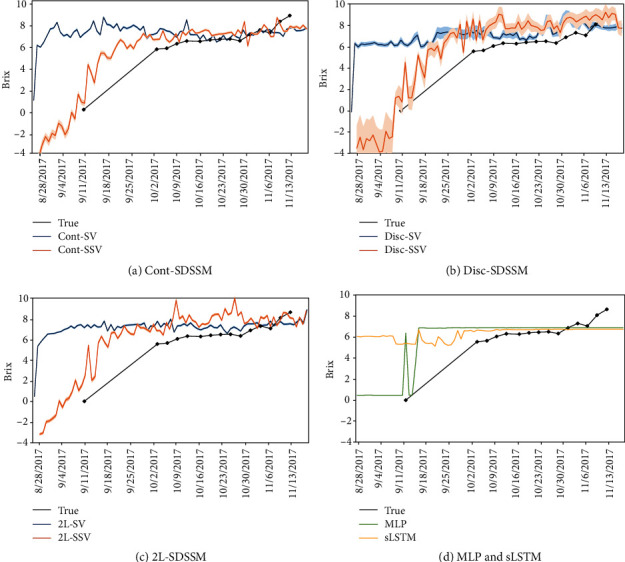
True and estimated values of the sugar content (brix) with the standard deviations for supervised SDSSMs, semisupervised SDSSMs, MLP, and sLSTM.

Figure 6 shows the true values, estimated values, and standard deviations of estimated values of time-series sugar content in the area 10 output by supervised SDSSMs, semisupervised SDSSMs, MLP, and sLSTM trained on dataset pattern A. The standard deviations of Cont-SDSSM and 2L-SDSSM are so low that they are not clearly visible. The results show that our semisupervised approach (Cont-SSV, Disc-SSV, and 2L-SSV) estimates the sugar contents better because of the improved generalization performance. Although there are few sugar content data before October (the other dataset patterns show a similar tendency because we collected sugar content data in the same manner over the entire area), the semisupervised SDSSMs clearly identify the variation patterns in other periods with their effective use of unlabeled data. From middle October to late October in Figure 6, although each supervised SDSSM (Cont-SV, Disc-SV, and 2L-SV) appears to be able to estimate better compared to the semisupervised SDSSMs, each supervised SDSSM's RMSE in Figure 5 is higher than that of each semisupervised SDSSM.

SDSSMs also output the variance and the estimation values. The averages of the standard deviations of the estimated values for all test plots in Table 2 are approximately 0.076 for Cont-SV, 0.28 for Disc-SV, 0.082 for 2L-SV, 0.17 for Cont-SSV, 1.04 for Disc-SSV, and 0.12 for 2L-SSV. In particular, the standard deviation of Disc-SSV is larger than those of the others, as clearly shown in Figure 6. A large standard deviation indicates output instability in the estimation for sugar content. The instability, however, is not a significant problem in a model for model-based RL when the standard deviation and estimation error are positively correlated. This is because the agent of the model-based RL explores an environmental model while considering the uncertainty of the estimation based on the standard deviation of the estimation value. Therefore, estimating the uncertainty correctly allows the agent to learn efficiently. MLP and LSTM look like they produce the same tendency as the supervised SDSSMS, and the estimated values in the period during October are relatively close to the true values, resulting in small errors, as shown in Figure 6(d). Considering only the numerical values, the MLP and LSTM look like they produce higher estimates than the supervised SDSSMs. However, as can be seen from Figure 6(d), the MLP and LSTM are overfitted to a particular dataset in the period after October even though dropout was applied in both cases, and the generalization performance is low.

**Figure 7:**
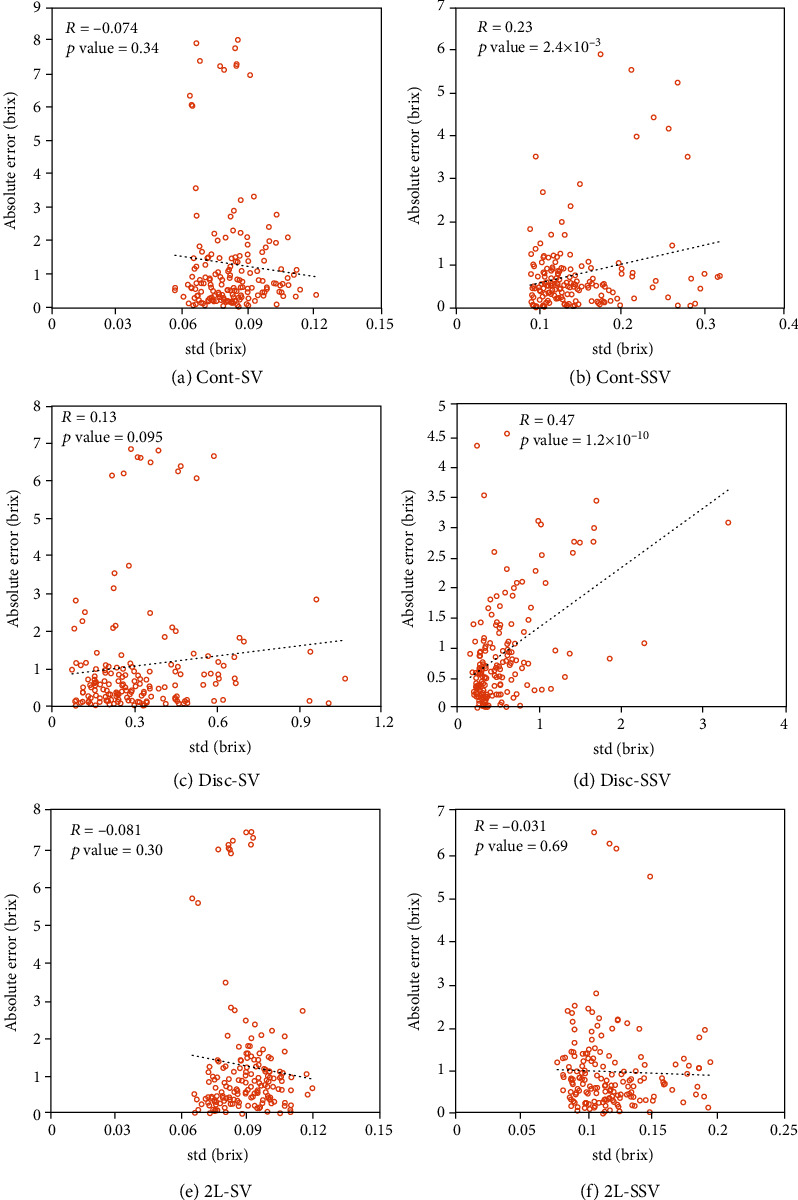
Scatter plots of standard deviations and absolute errors of supervised SDSSMs and semisupervised SDSSMs.

Figure 7 shows scatter plots of the standard deviations and the absolute errors for the compared models with the test dataset of pattern A in Table 1. Our proposed method is based on a generative model, which outputs both estimation values and standard deviations. Therefore, our proposed methods can evaluate the uncertainty of estimates by using the standard deviation. These results indicate that Cont-SSV and Disc-SSV significantly improve the correlation coefficient compared to supervised SDSSMs such as Cont-SV and Disc-SV. The correlation coefficient of 2L-SSV is still negative and is likely to cause incorrect exploration, although 2L-SSV slightly improves the correlation coefficient compared to 2L-SV. Conceivably, the significantly high correlation of 0.47 for Disc-SSV demonstrates that its standard deviations can assist agents to better seek an appropriate model and promote learning of the optimal control to achieve high sugar content.

**Figure 8:**
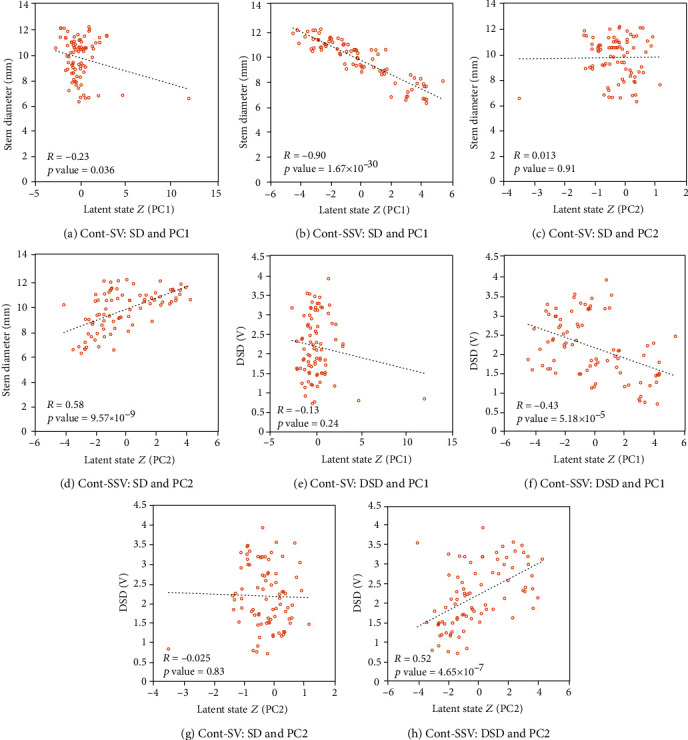
Scatter plots showing principal components and stem diameter or DSD.

Figure 8 shows the principal component and stem diameter or the difference in stem diameter (DSD) in the model learned using the dataset of pattern A in Table 1 as a scatter diagram on the *x*-axis and *y*-axis, respectively. The DSD is one of the water stress indicators and is expressed as the difference between the maximum stem diameter (SD) observed thus far and the current stem diameter (SD_*i*_) as follows: DSD_*i*_ = max(SD_0_, ⋯, SD_*i*_) − SD_*i*_. The maximum value continuously updates with plant growth. By calculating the decrease from the maximum stem diameter, the variation due to plant growth is ignored, and only the amount of water stress can be quantified from the stem diameter. This figure demonstrates that each principal component of the semisupervised Cont-SSV has a higher correlation with the stem diameter and DSD compared with Cont-SV. In particular, in Cont-SSV, the stem diameter has a significantly high correlation of approximately -0.9 with the first component, as shown in Figure 8(b), and DSD has a significantly high correlation of approximately 0.52 with the second component, as shown in Figure 8(h). This result suggests that Cont-SSV represents both plant growth and plant water content in the latent space owing to the reasonable inference achieved by using two observation variables sharing latent variables in our semisupervised learning model.

Additionally, the latent space is represented as a linear combination of these two plant states. This result confirms the assumption that the two types of latent variables are independent of each other. Cont-SSV and Disc-SSV have different natures, and Cont-SSV has better estimation accuracy, but Disc-SSV estimates the uncertainty better.

The results indicate that our three types of proposed models (Cont-SSV, Disc-SSV, and 2L-SSV) work better than the same models with supervised learning and other typical deep neural networks. In particular, Cont-SSV has good potential to estimate sugar content with high accuracy and valid uncertainty. Considering the appropriate representation of the latent states, it is believed that Cont-SSV will perform well as an environmental model of model-based RL for the optimal control of sugar content.

## 4. Conclusion

We have proposed a novel plant growth model using a semisupervised deep state-space model (SDSSM) for model-based reinforcement learning to determine the optimal control of sugar content. There have been several studies on tomato growth modeling [42, 43], but we could not find any similar study for modeling time-series tomato sugar content. SDSSM is a sequential deep generative model that uses structured variational inference to model the slow dynamics of living organisms (such as plant growth). In particular, SDSSM was trained using our semisupervised learning method that complementarily infers the latent states by introducing two observation variables to efficiently utilize sugar content data which is difficult to collect.

Additionally, we designed three types of SDSSMs under different assumptions regarding each latent space. The experimental results demonstrated that the introduction of two observation variables sharing latent variables improved the generalization performance and enabled all SDSSMs to track the variation of sugar content appropriately. Moreover, tomatoes grown during the experiment had a maximum brix rating of 10.73 and minimum brix rating of 4.67. The average brix rating was 6.81. The highest accuracy model is 0.78 in MAE; thus, our model has a potential to estimate time-series sugar content variation with high accuracy.

We have designed a combined model (2L-SDSSM); however, the combined model was not the highest accuracy model. Therefore, we still need to consider other ways to combine the two models more appropriately, i.e., assuming the independence of two latent states. In a future study, we intend to improve the 2L-SDSSM which is the combination of two different latent variables. Furthermore, we will improve time-series data (sensor data of the temperature, humidity, solar radiation, CO_2_ concentration, stem diameter, and plant growth) in a greenhouse different from that used in this study. We will continue to verify the performance of our model by comparing our model with typical machine learning and typical deep neural networks.

## Figures and Tables

**Figure 9:**
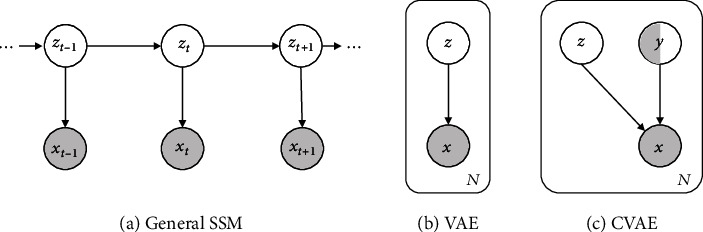
Graphical models of SSM, VAE, and CVAE.

**Table 1:** Variables in the proposed models.

Variable	Cont-SDSSM	Disc-SDSSM	2L-SDSSM
*x*_*t*_	Sugar content	Sugar content	Sugar content
*y*_*t*_	Stem diameter	Stem diameter	Stem diameter
**u**_**t**_	Temperature, solar radiation, VPD, elapsed date^1^, accumulated temperature^2^	Elapsed date^1^, accumulated temperature^2^	Temperature, solar radiation, VPD
**v**_**t**_	—	—	Elapsed date^1^, accumulated temperature^2^
**s**_**t**_	CO_2_ concentration, solar radiation, step ID	CO_2_ concentration, solar radiation, step ID	CO_2_ concentration, solar radiation, step ID

^1^Number of days after flowering. ^2^Summation of daily temperatures from the flowering date to the present.

**Table 2:** Data used for cross-validation.

Dataset pattern	Training (data size (labeled size) (cultivation bed no.))	Validation (data size (labeled size) (cultivation bed no.))	Test (data size (labeled size) (cultivation bed no.))
A	2,241 (382) (3, 4, 5, 6, 7, 11, 13, 14, 15)	747 (123) (8, 12, 16)	996 (167) (1, 2, 9, 10)
B	2,241 (345) (1, 5, 7, 8, 9, 13, 14, 15, 16)	747 (131) (2, 6, 10)	996 (154) (3, 4, 11, 12)
C	2,241 (361) (1, 2, 3, 4, 7, 9, 10, 11, 15)	747 (123) (8, 12, 16)	996 (189) (5, 6, 13, 14)
D	2,241 (381) (1, 3, 4, 5, 9, 11, 12, 13, 14)	747 (131) (2, 6, 10)	996 (161) (7, 8, 15, 16)

## Appendix

### A. Plant Growth Model Design

#### A.1 State-Space Model

The state-space model (SSM) is a generative model that represents time series based on two types of models: one is the system model and the other is the observation model. SSM models a sequence of observed variables *x*_1_, *x*_2_, ⋯, *x*_*T*_ and a corresponding sequence of latent variables *z*_1_, *z*_2_, ⋯, *z*_*T*_ at each time step *t* (*t* = 1, 2, ⋯, *T*). The probabilistic model is shown in Figure 9(a), and a general expression of the model is

(A.1) $$\begin{array}{c} z_{t}\sim p\left(z_{t}\;|\;z_{t-1},\theta \right),\left(\text{system model}\right),\\ x_{t}\sim p\left(x_{t}\;|\;z_{t},\varphi \right),\left(\text{observation model}\right), \end{array}$$

where the system model is the conditional probability distribution of the latent state *z*_*t*_ conditioned on the previous latent state *z*_*t*−1_ and the observation model is the conditional probability distribution of *x*_*t*_ conditioned on the corresponding latent state *z*_*t*_ at time step *t*. The variables *θ* and *φ* denote the parameter vectors of the system model and the observation model, respectively. The system model is initialized by *z*_0_ = *p*(*z*_0_). We use available arbitrary probability distributions in both the system model and the observation model. Some SSMs with specific distributions and constraints have unique names. For example, one of the simplest models, called a linear Gaussian model (LGM) [44], can be written mathematically as

(A.2) $$\begin{array}{c} z_{t+1}=A_{t}z_{t}+\epsilon_{t},\epsilon_{t}\sim N\left(0,Q\right),\left(\text{system model}\right),\\ x_{t}=B_{t}z_{\text{t}}+\omega_{t},\omega_{t}\sim N\left(0,R\right),\left(\text{observation model}\right), \end{array}$$

where vector *ϵ*_*t*_ and vector *ω*_*t*_ are random variables representing the state and observation noises, respectively. In addition, *x*_*t*_ and *z*_*t*_ are vectors. Both of these noises are independent of each other, the corresponding latent state *z*_*t*_, and the conditional probability distribution of *x*_*t*_. Both of these noise sources are a Gaussian distribution with zero covariance matrix representing the mean *Q* and *R*, each independent of the time step. *A*_*t*_ and *B*_*t*_ denote coefficient matrices. LGMs can fit with time-series data and are utilized for many applications whose observations and latent states have a linear transition and a normal distribution, respectively.

Additionally, there are methods available to model time-series data with nonlinear transitions, e.g., the extended Kalman filter [45] and the quadrature particle filter [46]. Raiko et al. [47] and Valpola and Karhunen [22] attempted to estimate an intractable posterior of the complex generative model using nonlinear dynamic factor analysis, which is impractical for large-scale datasets owing to the inherent quadratic scale regarding observed dimensions. Recently, Kingma and Welling [29] introduced stochastic gradient variational Bayes (SGVB) and a learning algorithm named a variational autoencoder (VAE) to obtain a tractable posterior.

#### A.2 Variational Autoencoder

The variational autoencoder (VAE) [22] is a deep generative model for nonsequential data. The parameters are optimized via SGVB [23], and the probabilistic model is shown in Figure 9(b). In particular, conditional VAE (CVAE), as shown in Figure 9(c), is a typical deep generative model trained by semisupervised learning [21]. The *N* in the plate (the part surrounded by the frame) shown in Figure 9 means that*N*nodes are omitted and only the representative nodes are shown in Figures 9(b) and 9(c). The details will be discussed later in this section. In the VAE, the generative process can be written mathematically as

(A.3) $$\begin{array}{c} z\sim p\left(z\right),\\ x\sim p_{\theta }\left(x\;|\;z\right). \end{array}$$

The latent variable *z* is obtained from a prior *p*(*z*). The observed variable *x* is drawn from a probability distribution *p*_*θ*_(*x* | *z*) conditioned on *z* with the parameter vector *θ*, and the posterior *p*_*θ*_(*z* | *x*) is assumed to be intractable. The parameter is estimated simultaneously with the latent states through maximization of the following marginal log-likelihood:

(A.4) $$\begin{array}{c} \text{Log}p_{\theta }\left(x\right)=\log\int p_{\theta }\left(x\;|\;z\right)p\left(z\right)dz. \end{array}$$

When the posterior is intractable, the standard expectation-maximization (EM) algorithm and variational inference do not work well because the EM algorithm needs to compute the posterior and variational inference requires closed-form solutions of the expectations of the joint probability density function. Additionally, sampling-based EM algorithms require sufficient time to obtain the posterior when the data size is large. In SGVB, the approximate posterior *q*_*φ*_(*z* | *x*) with parameter *φ* is introduced, and we consider the following evidence lower bound (ELBO) *ℒ*(*x*; *θ*, *φ*) as would be the case for the variational inference. ELBO is an objective function used to optimize the parameters *θ* and *φ*.

(A.5) $$\begin{array}{c} L\left(x;\theta ,\varphi \right)=E_{q_{\varphi }\left(z\;|\;x\right)}\left[\log p_{\theta }\left(x\;|\;z\right)\right]-\text{KL}\left(q_{\varphi }\left(z\;|\;x\right)\left\|p\left(z\right)\right.\right). \end{array}$$

KL denotes a Kullback–Leibler divergence. In addition, parameter *φ* denotes the parameter of *q*_*φ*_(*z* | *x*) that approximates *p*_*θ*_(*x*). In fact, in the VAE, *φ* represents the neural network's weights and bias, and these parameters are optimized via back-propagation. The difference in variational inference is the use of differentiable Monte Carlo expectations instead of applying the mean-field assumption. Formally, the reformulation of the ELBO is as follows:

(A.6) $$\begin{array}{c} L\left(x;\theta ,\varphi \right)≅\frac{1}{L}\overset{L}{\underset{l=1}{\sum }}\log p_{\theta }\left(x\;|\;z^{\left(l\right)}\right)-\text{KL}\left(q_{\varphi }\left(z\;|\;x\right)\left\|p\left(z\right)\right.\right), \end{array}$$

where *z* is sampled via a reparameterization trick (*z*^(*l*)^ = *μ* + *σε*^(*l*)^ and *ϵ*^(*l*)^ ~ *N*(0, *I*)) to acquire a differentiable ELBO instead of sampling from the posterior *q*_*φ*_(*z* | *x*) (which is not differentiable with respect to *φ*). Specifically, the reparameterization trick represents a sampling z ~ *q*_*φ*_(*z* | *x*) as a deterministic transformation *g*_*φ*_(*ϵ*, *x*) by adding a random noise *ϵ* ~ *p*(*ϵ*) to input *x*. By using the reparameterization trick, the expectation term can be written mathematically as *E*_*q*_*φ*_(*z* | *x*)_[*f*(*z*)]≅1/*L*∑_*l*=1_^*L*^*f*(*g*_*φ*_(*ϵ*^(*l*)^, *x*)) where *f*(*z*) = log*p*_*θ*_(*x* | *z*) and *ϵ*^(*l*)^ ~ *p*(*ϵ*). This expectation is differentiable with respect to *θ* and *φ*. For example, when a latent variable *z* is distributed according to a Gaussian distribution *N*(*z* | *μ*, *σ*^2^), the ELBO is as follows:

(A.7) $$\begin{array}{c} E_{q_{\varphi }\left(z\;|\;x\right)}\left[\log p_{\theta }\left(x\;|\;z\right)\right]≅\frac{1}{L}\overset{L}{\underset{l=1}{\sum }}\log p_{\theta }\left(x\;|\;z^{\left(l\right)}\right). \end{array}$$

The likelihood *p*_*θ*_(*x* | *z*) and posterior *q*_*φ*_(*z* | *x*) are represented by neural networks, and the parameters are optimized via back-propagation.
